# Efficient Method for the Concentration Determination of Fmoc Groups Incorporated in the Core-Shell Materials by Fmoc–Glycine

**DOI:** 10.3390/molecules25173983

**Published:** 2020-09-01

**Authors:** Elżbieta Szczepańska, Beata Grobelna, Jacek Ryl, Amanda Kulpa, Tadeusz Ossowski, Paweł Niedziałkowski

**Affiliations:** 1 Department of Analytical Chemistry, Faculty of Chemistry, University of Gdansk, Wita Stwosza 63, 80-308 Gdansk, Poland; elzbieta.szczepanska@phdstud.ug.edu.pl (E.S.); beata.grobelna@ug.edu.pl (B.G.); amanda.kulpa@phdstud.ug.edu.pl (A.K.); tadeusz.ossowski@ug.edu.pl (T.O.); 2 Corrosion and Materials Engineering, Department of Electrochemistry, Faculty of Chemistry, Gdansk University of Technology, Narutowicza 11/12, 80-233 Gdansk, Poland; jacek.ryl@pg.edu.pl

**Keywords:** loading of amino groups, Fmoc–Gly–OH, TiO_2_@SiO_2_ core-shell, dibenzofulvene–piperidine adduct, nanoparticles

## Abstract

In this paper, we described the synthesis procedure of TiO_2_@SiO_2_ core-shell modified with 3-(aminopropyl)trimethoxysilane (APTMS). The chemical attachment of Fmoc–glycine (Fmoc–Gly–OH) at the surface of the core-shell structure was performed to determine the amount of active amino groups on the basis of the amount of Fmoc group calculation. We characterized nanostructures using various methods: transmission electron microscope (TEM), scanning electron microscopy (SEM), Fourier-transform infrared spectroscopy (FTIR), thermogravimetric analysis (TGA) and X-ray photoelectron spectroscopy (XPS) to confirm the modification effectiveness. The ultraviolet-visible spectroscopy (UV-vis) measurement was adopted for the quantitative determination of amino groups present on the TiO_2_@SiO_2_ core-shell surface by determination of Fmoc substitution. The nanomaterials were functionalized by Fmoc–Gly–OH and then the fluorenylmethyloxycarbonyl (Fmoc) group was cleaved using 20% (*v/v*) solution of piperidine in DMF. This reaction led to the formation of a dibenzofulvene–piperidine adduct enabling the estimation of free Fmoc groups by measurement the maximum absorption at 289 and 301 nm using UV-vis spectroscopy. The calculations of Fmoc loading on core-shell materials was performed using different molar absorption coefficient: 5800 and 6089 dm^3^ × mol^−1^ × cm^−1^ for λ = 289 nm and both 7800 and 8021 dm^3^ × mol^−1^ × cm^−1^ for λ = 301 nm. The obtained results indicate that amount of Fmoc groups present on TiO_2_@SiO_2_–(CH_2_)_3_–NH_2_ was calculated at 6 to 9 µmol/g. Furthermore, all measurements were compared with Fmoc–Gly–OH used as the model sample.

## 1. Introduction

Titanium dioxide (TiO_2_) possesses many advantages, is nontoxic, highly efficient, photocatalytically stable and cost-effective [1,2]. Therefore, this compound has been extensively used in many fields. Recently, TiO_2_ has found several applications, including the development of new fluorescent materials [3,4], UV filters for cosmetics and paint pigments, [5] or photocatalysts used for water and air purification [6].

Nowadays, various metal nanoparticles such as silver, gold or titanium are widely used not only in industry, but also in nanotechnology and other various fields of research [7,8]. The titanium dioxide nanoparticles (TiO_2_ NPs) are utilized in many fields of industry [9]. Nevertheless, one of the biggest drawbacks of TiO_2_ NPs is the tendency to agglomerate. This limitation has been solved by the deposition of TiO_2_ NPs on surfaces of other materials such as silica (SiO_2_) resulting in the formation of TiO_2_@SiO_2_ core-shell particles. The SiO_2_ coating of NPs provides the colloidal stability, but the presence of silanol groups on the NPs surface contribute to the possible additional modification by various functional groups such as: alkyl [10], hydroxyl [11], thiol (-SH) [12,13], carboxyl (-COOH) [14], amine (-NH_2_) [15], EDTA derivatives [16] or other nanoparticles and ions [17,18].

3-(Aminopropyl)trimethoxysilane (APTES) is one of the most popular agents used for modifications of various nanoparticles such as TiO_2_ NPs [19], Fe_3_O_4_ [20], glass [21] or solid electrodes [22]. The presence of amino silane after reaction with hydroxyl-terminated surface makes it possible to obtain a new material with terminal amino groups. Consequently, these amino groups enable the immobilization of new biomolecules, such as proteins [23], enzymes [24], antibodies [25,26] or DNA samples [27].

The mechanism of NPs modification by alkoxysilanes consists of two steps. The first process is associated with the hydrolysis of silane groups of used modification agent which leads to the formation of alcohol and active alkoxysilane derivative. The second step leads to the condensation of organosilanols with hydroxyl groups present on the modified NPs surface. In this step, the condensation process also occurs [28]. The mechanism of action for APTES is based on the reaction with the hydroxyl group of the oxidized surface by SN2 exchange and loss of ethanol. The same reaction is observed in the case of APTMS usage, but during the surface covering the methanol is formed as a byproduct. The detailed studies of APTES hydrolysis and then condensation. including various details such as time reaction, concentration, temperature or pH is described elsewhere [25,29,30,31].

In the synthesis of modified nanoparticles—as well as in immobilization of biomolecules onto the surface—an important parameter characterizing the modified material is the estimation of the number of functional groups deposited on the surface to assess further modifications or material analyses. Determination of the amino group concentration is the most important parameter after modification by amino silanes. Estimation of amino groups can be obtained by various analytical methods, including acid–base back titration or reaction [32], conductometric titrations [33], NMR [34] or ζ-potential and direct measurements [35], thermogravimetric analysis [36] or X-ray photoelectron spectroscopy (XPS) analysis [37].

Additionally, amino functional groups can be estimated using various analytical methods after chemical reaction described above. The absorbance measurements after reaction with fluorescent rhodamine B isothiocyanate [32], the ninhydrin assay, as well as 4-nitrobenzaldehyde assay and NMR measurements are exceptionally useful for the determination of the amino functional groups [38]. Nevertheless, all of these methods show various sensitivities and advantages, but the main limitation of the methods listed above is the small amount of NPs for analyses.

In this work, we obtained TiO_2_@SiO_2_ core-shell by Stöber method. The main advantage of the Stöber method based on sol–gel chemistry is simplicity, wide availability of reagents and possibility of obtaining three-dimensional materials such as core shell nanoparticles in a one-pot synthesis. This method allow to obtain nanostructures with nano to micrometers size [39].

Subsequently, obtained TiO_2_@SiO_2_ core-shell was modified using (3-aminopropyl)trimethoxysilane (APTMS) to determine Fmoc groups concentration, what enabled the estimation of the amino group presence on the surface. Nowadays, the estimation of amino group presence on the surface of amino-modified nanomaterials is very problematic and requires much effort and instruments. Therefore, the nanoparticles were functionalized by Fmoc–Gly–OH and then Fmoc group was cleaved using 20% (*v*/*v*) piperidine solution in DMF. This reaction led to the formation of dibenzofulvene–piperidine adduct, used for the estimation of free amino groups by the measurement of the maximum absorption at 289 and 301 nm. The obtained results indicate that amount of Fmoc groups present on TiO_2_@SiO_2_–(CH_2_)_3_–NH_2_ was calculated at 6 to 9 µmol/g. All measurements were also compared with the solution of Fmoc–Gly–OH used as the model sample.

## 2. Results and Discussion

### 2.1. Synthesis

**Scheme 1:**
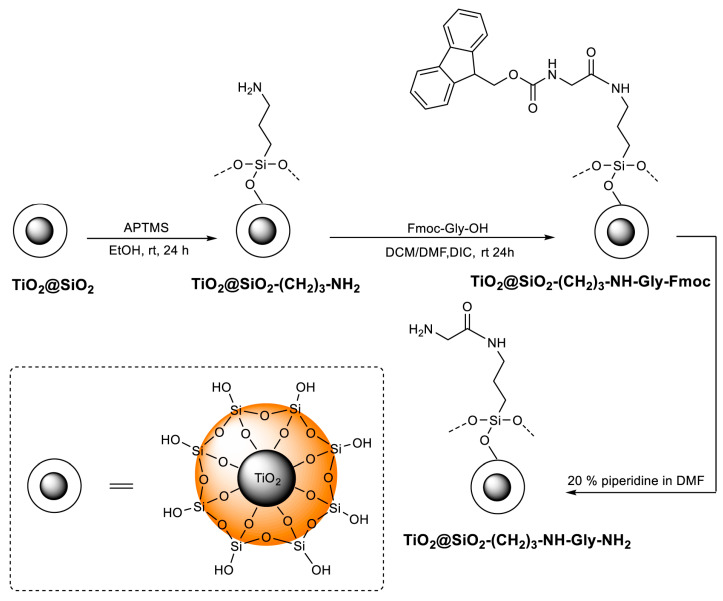
Synthesis of TiO_2_@SiO_2_ with (3-aminopropyl)trimethoxysilane (APTMS), Fmoc–Gly–OH and cleavage by piperidine in 20% (*v*/*v*) solution in DMF.

The synthesis and modification steps of TiO_2_ NPs are presented on Scheme 1. The TiO_2_ NPs were obtained in the reaction of titanium (IV) isopropoxide (TTIP) used as a titanium source and SDS as a surfactant by the microemulsion method previously described in Yuenyongsuwan procedure [40].

TiO_2_ NPs obtained using this method were used for further modifications. The TiO_2_@SiO_2_ core-shell structures were synthesized using Stöber method [41] with tetraethyl orthosilicate (TEOS) in the solution of pH = 9 adjusted by ammonia hydroxide.

In described Stöber method the core shell nanostructures are formed by hydrolysis and condensation of TEOS in ethanol and water catalyzed by ammonia hydroxide at room temperature [39,42].

The TiO_2_@SiO_2_ core-shell structures in this work were obtained by the adoption of the method described for the preparation of Ag@SiO_2_ core-shell nanoparticles [43].

**Figure 1:**
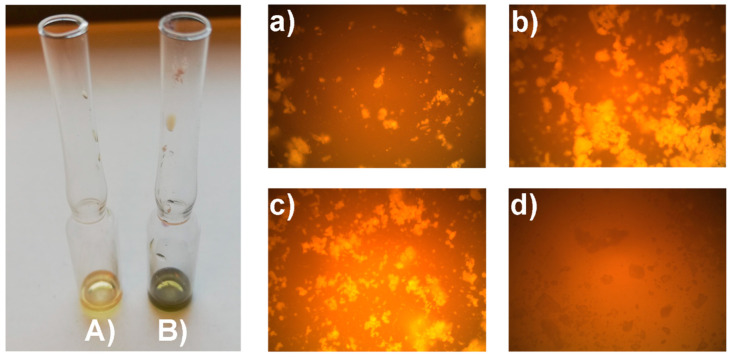
Chloranil test performed for (**A**) TiO_2_@SiO_2_ and (**B**) TiO_2_@SiO_2_–(CH_2_)_3_–NH_2_. The microscopic magnification for core-shell TiO_2_@SiO_2_ nanostructures (**a**) before and (**b**) after chloranil test and for TiO_2_@SiO_2_–(CH_2_)_3_–NH_2_ NPs (**c**) before and (**d**) after chloranil test.

In the next step, the obtained core-shell TiO_2_@SiO_2_ nanostructures were modified by an aminosilanization process using the post-grafting method with 3-(aminopropyl)trimethoxysilane (APTMS) as the reagent [44]. After the completion of the reaction, the presence of amino groups was confirmed using chloranil test performed on TiO_2_@SiO_2_–(CH_2_)_3_–NH_2_ with microscopic magnification [45]. Figure 1 shows the comparison of chloranil test performed for TiO_2_@SiO_2_ and TiO_2_@SiO_2_–(CH_2_)_3_–NH_2_ and its microscopic magnification. The chloranil test used in our experiments was consist of equally volumetric amounts of 2% solutions acetaldehyde in DMF and 2% solution of p-chloranil dissolved in DMF. After 15 min the change of colors from white to greenish blue was observed for TiO_2_@SiO_2_–(CH_2_)_3_–NH_2_ NPs indicating the presence of amino groups. On the other hand, the change of color for core-shell TiO_2_@SiO_2_ nanostructure were not observed due to the fact of absence of amino group.

Subsequently, the modified TiO_2_@SiO_2_–(CH_2_)_3_–NH_2_ NPs were used for coupling with Fmoc–glycine (Fmoc–Gly–OH). This reaction was performed in a solution consisting of methylene chloride: DMF, 1:1, (*v*/*v*) in the presence of 4-dimethylaminopyridine (DMAP) and diisopropylcarbodiimide (DIC) obtaining TiO_2_@SiO_2_–(CH_2_)_3_–NH–Gly–Fmoc. This procedure is used in peptide chemistry for the attachment of amino acids for resins calculations [46]. The Fmoc deprotection was performed using 20% (*v*/*v*) piperidine in DMF commonly used in the solid-phase peptide synthesis [47].

### 2.2. Analysis of Obtained Nanoparticles

**Figure 2:**
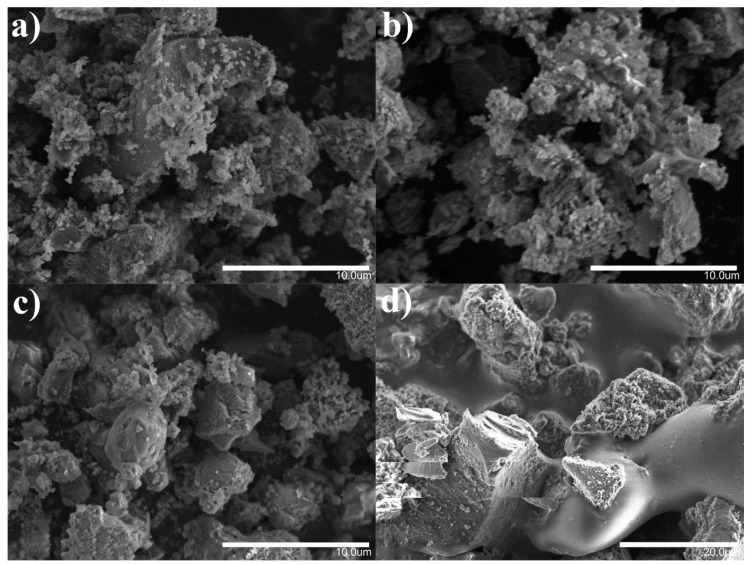
Scanning electron microscopy (SEM) images of (**a**) TiO_2_, (**b**) TiO_2_@SiO_2_, (**c**) TiO_2_@SiO_2_–(CH_2_)_3_–NH_2_ and (**d**) TiO_2_@SiO_2_–(CH_2_)_3_–NH–Gly–Fmoc.

TiO_2_ nanostructures as well as other studied nanoparticles were characterized using scanning electron microscopy (SEM). SEM images presented in Figure 2 show the presence of nanoparticles in the highly aggregated state.

**Figure 3:**
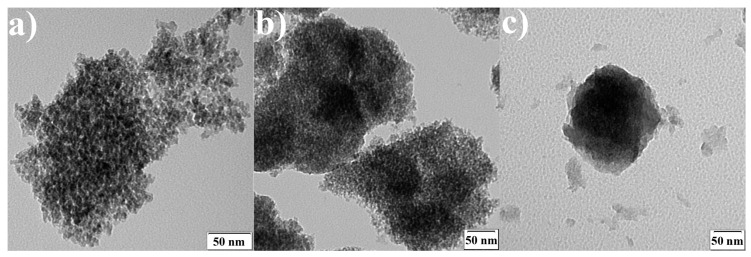
Transmission electron microscopy (TEM) images of (**a**) TiO_2_, (**b**) TiO_2_@SiO_2_ and (**c**) TiO_2_@SiO_2_–(CH_2_)_3_–NH_2_.

No significant differences between the nanoparticles are not observed after each step of modifications. Therefore, in order to receive more precise visualization of studied nanoparticles surface the TEM images were obtained (Figure 3). SEM analysis of all nanoparticles were performed for powders, while TEM images were obtained for the nanoparticle suspensions which influence on the behavior of studied nanostructures.

The TEM images of three kinds of nanostructures—TiO_2_, TiO_2_@SiO_2_, and TiO_2_@SiO_2_–(CH_2_)_3_–NH_2_—are presented in Figure 3. The structure of TiO_2_@SiO_2_–(CH_2_)_3_–NH–Gly–Fmoc is strikingly similar to TiO_2_@SiO_2_–(CH_2_)_3_–NH_2_, thus these images are not presented.

All nanoparticles have a spherical shape, but they significantly differ in the aggregation levels. We can observe that aggregation into larger clusters decreased with the modification degree. The highest aggregation is observed in the decreasing order for TiO_2_@SiO_2_–(CH_2_)_3_–NH_2_, TiO_2_@SiO_2_ and TiO_2_. The covering of TiO_2_ nanoparticles by silica core-shell structures and further modification by APTMS prevent the aggregation process [48,49].

The average size of nanoparticles is about 10 nm for TiO_2_, TiO_2_@SiO_2_ and 100 nm for TiO_2_@SiO_2_–(CH_2_)_3_–NH_2_.

**Figure 4:**
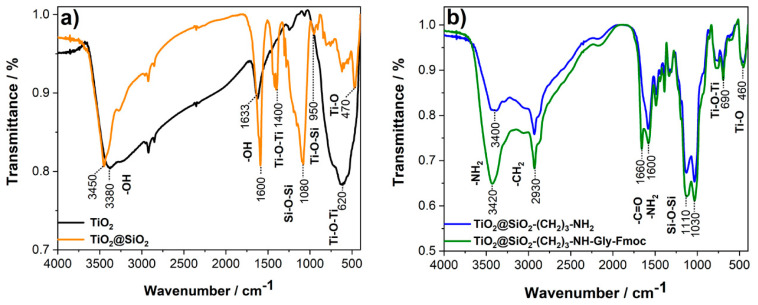
Fourier-transform infrared spectra of (**a**) TiO_2_, TiO_2_@SiO_2_ and (**b**) TiO_2_@SiO_2_–(CH_2_)_3_–NH_2_, TiO_2_@SiO_2_–(CH_2_)_3_–NH–Gly–Fmoc.

Figure 4 shows a sequence of FTIR spectra for TiO_2_, TiO_2_@SiO_2_, TiO_2_@SiO_2_–(CH_2_)_3_–NH_2_ and TiO_2_@SiO_2_–(CH_2_)_3_–NH–Gly–Fmoc the characteristic bands of TiO_2_ were observed Figure 4a. The broadband region located at the 3380 cm^−1^ is related to the stretching vibration of the hydroxyl group (-OH). The band at the 1633 cm^−1^ is assigned to bending modes of Ti–OH water. Additionally, the last band located at 620 cm^−1^ range represents the stretching vibrations of Ti–O–Ti binding [50,51]. However, the FTIR spectrum of TiO_2_@SiO_2_—presented in Figure 4a—shows more absorption bands in comparison with the absorption bands observed for the titanium dioxide. The characteristic bands at 3450 cm^−1^ and 1600 cm^−1^ in the spectra are attributed to the stretching vibration and bending vibration of hydroxyl groups. The band at 1400 cm^−1^ was assigned as the Ti–O–Ti vibration [52]. The peaks at 1080 cm^−1^, 950 cm^−1^ and 470 cm^−1^ correspond to the stretching vibrations for Si–O–Si, Ti–O–Si and Ti–O, respectively [53]. Figure 4b presents TiO_2_@SiO_2_–(CH_2_)_3_–NH_2_ particles. The peak at 3400 cm^−1^ is associated with -NH_2_ stretching vibrations and the peaks at 2930 cm^−1^ are assigned to the stretching vibrations of -CH_2_ groups. The absorption band at 1600 cm^−1^ is associated with the presence of -NH_2_ deformation mode [54]. Furthermore, the absorption peaks at 1130 and 1030 cm^−1^ belong to Si–O–Si, peak at 690 cm^−1^ was assigned to the stretching vibration of the Ti–O–Ti bond, the Ti–O group is observed at 460 cm^−1^. For TiO_2_@SiO_2_–(CH_2_)_3_–NH–Gly–Fmoc (Figure 4b) similar bands are observed as in the case of TiO_2_@SiO_2_–(CH_2_)_3_–NH_2_. One of the most characteristic peaks is observed at 1660 cm^−1^ which presents stretching groups combined with carbonyl groups (-C=O) [55]. This analysis confirmed the modification of TiO_2_ with silica, amino groups and Fmoc–Gly–OH.

**Figure 5:**
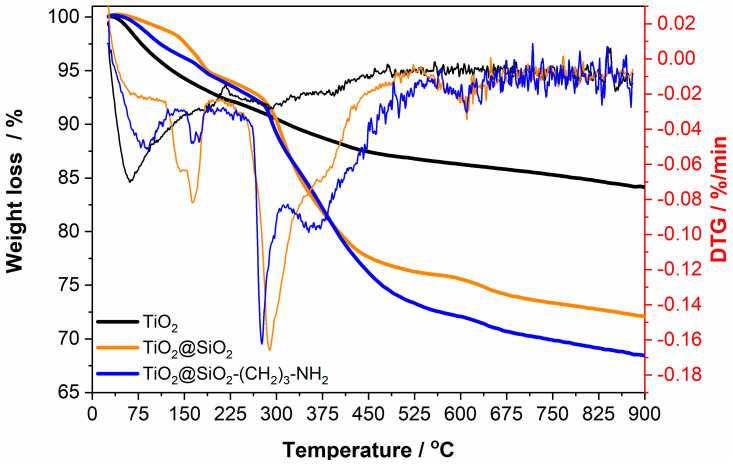
Thermogravimetric (TG) and thermogravimetric derivative (DTG) curves for TiO_2_, TiO_2_@SiO_2_ and TiO_2_@SiO_2_–(CH_2_)_3_–NH_2_.

Thermogravimetric analysis (TGA) and derivative thermogravimetric analysis (DTG) for TiO_2_, TiO_2_@SiO_2_ and TiO_2_@SiO_2_–(CH_2_)_3_–NH_2_ is presented at Figure 5. For TiO_2_ NPs, the 7.92% weight loss is observed between 65 and 200 °C, this process is probably associated with physical desorption of water on the surface [49]. Next visible changes are observed within the range 300 to 410 °C with the weight loss of 5.28% attributed to the reduction of hydroxyl groups.

For TiO_2_@SiO_2_ core-shell, 6.65% weight loss in the 350 to 500 °C range indicates the disintegration of hydroxyl groups in silanol moieties [56]. The range of 500 to 700 °C is characterized with the weight loss of 2.74% due to the degradation of -CH_2_CH_3_ alkyl group in TEOS [49].

In the case of TiO_2_@SiO_2_–(CH_2_)_3_–NH_2_ structure, the largest decrease in mass of 14.01% within 400 to 600 °C range was caused by the thermal decomposition of APTMS molecule.

**Figure 6:**
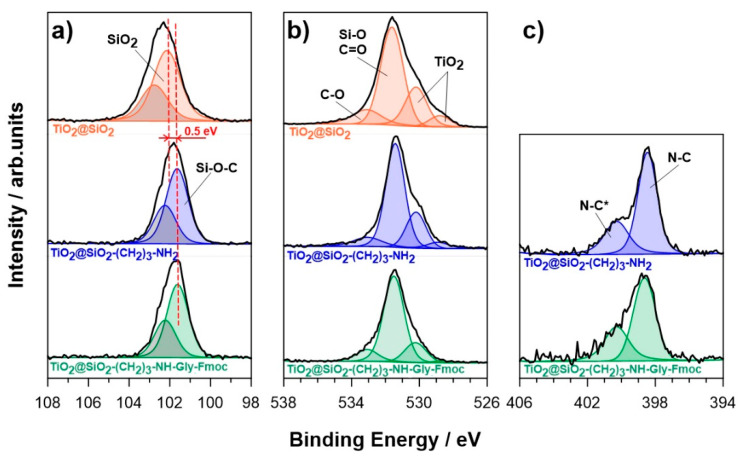
High-resolution XPS spectra for (**a**) Si2p, (**b**) O1s and (**c**) N1s recorded for each studied surface-modification and encapsulation step of TiO_2_@SiO_2_, together with the proposed deconvolution procedure.

Figure 6 reveals the changes in the surface chemistry of the nanoparticles upon consecutive functionalization steps during formation of the core-shell materials.

First, the successful formation of the TiO_2_@SiO_2_ particles was confirmed through the appearance of strong Si2p peak doublet at approx. 102.1 eV, which is typical for SiO_2_ monolayer surface coverage on TiO_2_ [57,58] (Figure 6a). The TiO_2_@SiO_2_ O1s reveals a complex nature, with multiple components characteristic for the SiO_2_ (531.6 eV), TiO_2_ (528.8 eV) and atomically mixed TiO_2_–SiO_2_ thin film (530.2 eV) [16,58], which are further modified by the presence of small amounts of adventitious carbon, carbonate and adsorbed water contamination resultant from the air exposure [59]. The observed SiO_2_:mixed:TiO_2_ ratio was 9.2:3.5:1. The detailed information regarding the deconvolution with the proposed model is summarized in Table 1.

The consecutive functionalization of the core-shell TiO_2_@SiO_2_–(CH_2_)_3_–NH_2_ with amino groups led to a negative shift of the Si2p peak doublet position by 0.5 eV due to the appearance of silicon oxycarbide Si–O–C interaction [60,61]. In parallel, the oxygen surface chemistry is significantly altered. The share of mixed TiO_2_–SiO_2_, SiO_2_, and TiO_2_ components drops approximately by a factor of 1.5. Last, but not least, a strong signal recorded in the N1s energy range revealed the presence of the amino -NH_2_ species (398.5 eV) [62,63]. The N1s signal is characterized by a complex structure, with a second, two times smaller component, which may be the effect of NH_3_^+^ protonation or deprotonation on NH-.

Finally, the incorporation of Fmoc–glycine on the surface of encapsulated TiO_2_@SiO_2_ nanoparticles only slightly changed the chemistry, based on XPS analyses. Further decay in the O1s signal strength from titanium and silicon oxides was observed, except for the component located at 531.6 eV, which source next to SiO_2_ is also C=O bonds in glycine [64,65]. Based on the XPS analyses of core level Si2p, O1s and N1s binding energy range (Figure 6a–c), the chemistry of SiO_2_ and NH_2_ species did not change significantly between TiO_2_@SiO_2_–(CH_2_)_3_–NH_2_ and TiO_2_@SiO_2_–(CH_2_)_3_–NH–Gly–Fmoc nanoparticles. Moreover, the TiO_2_ presence may no longer be recognized based on the Ti2p spectra.

### 2.3. Determination of Fmoc Group Loading on TiO_2_@SiO_2_–(CH_2_)_3_–NH–Gly–Fmoc Core-Shell Nanostructure

**Scheme 2:**
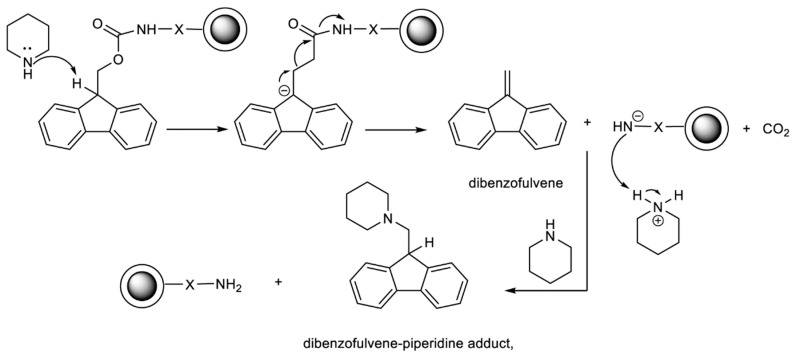
Mechanism of Fmoc group removal from solid support and formation of dibenzofulvene–piperidine adduct in the presence of piperidine.

Nowadays, the solid-phase peptide synthesis using Fmoc/*t*-Bu method is one of the most frequent strategies to obtain a peptide. This method is applied for the peptide synthesis on a solid support with amino acid derivatives containing a base-labile Fmoc group protecting the amino group and acid-labile side chains [66]. Since there is a wide distribution of Fmoc amino acid derivatives, the peptide synthesis using this strategy is particularly cost-effective and does not require the usage of toxic hydrogen fluoride [67]. Fmoc group during synthesis is usually removed in a weak basic conditions, in 20% of piperidine in DMF. The reaction mechanism of Fmoc cleavage from solid support presented in Scheme 2 in the first step is based on β-elimination [68]. Initially, the proton from the fluorenyl group at the 9-position is removed by piperidine with the formation of dibenzofulvene Scheme 2. In the next step, the presence of piperidine causes the formation of dibenzofulvene–piperidine adducts based on the Michael-type addition [69]. This reaction usually occurs in electron-donor polar solvents, such as DMF.

The reaction of Fmoc protected core-shell and piperidine led to the formation of piperidine-dibenzofulvene adduct. Due to the fact that this adduct is soluble in reaction solvents, one can use the spectrophotometric measurement to determine the concentration of Fmoc groups based on Beer–Lambert law. The determination of Fmoc groups loading at core-shell nanostructure enable the calculation of the amino group prevalence at amino core-shell structure before the modification by Fmoc–Gly–OH.

In this work, we adopted the method previously invented for the determination of first anchored Fmoc-protected amino acids on resins to measure the presence of amino groups on the core-shell structure. Both mechanism and determination of molar absorption coefficient (ε) for piperidine-dibenzofulvene in different solutions have been reported in many studies. Therefore, we decided to calculate and compare the loading of Fmoc groups based the on literature data using a different ε value both for core-shell structure, as well as for Fmoc–Gly–OH model sample.

**Figure 7:**
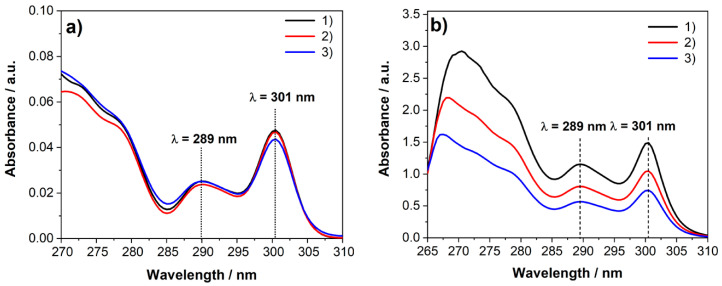
Absorption spectra with the concentrations in mol/dm^3^ of (**a**) TiO_2_@SiO_2_–(CH_2_)_3_–NH–Gly–Fmoc, (**b**) Fmoc–Gly–OH used as the model sample.

The spectrophotometric measurements performed for both TiO_2_@SiO_2_–(CH_2_)_3_–NH–Gly–Fmoc and Fmoc–Gly–OH are presented in Figure 7. In both graphs, two absorption maxima at 289 and 301-nm wavelengths are observed, corresponding to the presence of dibenzofulvene–piperidine adduct in the solution. The mechanism of the occurring reaction is presented in Scheme 2. The molar absorption coefficient (ε) was determined previously for this absorption maxima: ε (267 nm) = 17,500 dm^3^ × mol^−1^ × cm^−1^,ε (290 nm) = 5800 dm^3^ × mol^−1^ × cm^−1^ and ε (301 nm) 7800 dm^3^ × mol^−1^ × cm^−1^ in methylene chloride [70]. The similar value was also used for DMF solution: ε (266 nm) = 17,500 dm^3^ × mol^−1^ × cm^−1^, ε (289 nm) = 5800 dm^3^ × mol^−1^ × cm^−1^ and ε (300 nm) 7800 dm^3^ × mol^−1^ × cm^−1^ [71]. In many studies only one ε coefficient (301 nm) 7800 dm^3^ × mol^−1^ × cm^−1^ was used for the calculation of the loading of amino acids and other molecules on a resin [69,72,73,74,75].

Nevertheless, the detailed research on Fmoc-substituted resin at a different wavelength determined previously (ε = 6089 dm^3^ × mol^−1^ × cm^−1^) at 289,8 nm and (ε = 8021 dm^3^ × mol^−1^ × cm^−1^) at 301 nm [76] encouraged us to compare different ε coefficient of dibenzofulvene–piperidine in DMF for calculation of Fmoc groups on core-shell nanostructures and model sample (Fmoc–Gly–OH).

All measurements of adsorption spectra of dibenzofulvene–piperidine adduct were performed in triplicates. In the case of TiO_2_@SiO_2_–(CH_2_)_3_–NH–Gly–Fmoc as well as Fmoc–Gly–OH, two absorption maxima were observed at 289 nm and 301 nm Figure 7. In both cases also the absorption band was observed at 267 nm, but due to the fact of intensity differences, this band was not included in the calculations of Fmoc loading. The detailed data of the maximum absorbance value for the examined materials are presented in Table 2.

The estimation of the Fmoc group (Z) loading on the TiO_2_@SiO_2_–(CH_2_)_3_–NH–Gly–Fmoc core-shell nanostructure surface was calculated according to the equation presented in the paragraph 3.4. The comparison of all calculated loadings of the Fmoc group for both examined materials is presented in Table 3. The obtained data directly indicate that in the case of TiO_2_@SiO_2_–(CH_2_)_3_–NH–Gly–Fmoc the similar values of loading for Fmoc groups were obtained (6 µmol/g) taking into consideration the maximum band at λ = 289 nm and using both molar absorption coefficients: ε = 5800 and 6089 dm^3^ × mol^−1^ × cm^−1^. The calculation performed for 301-nm wavelength confirmed the relation presented above. Conducted calculations gave the similar values of loading for Fmoc groups at the core-shell nanostructure of about 8 or 9 µmol/g using molar absorption coefficient ε = 7800 and 8021 dm^3^ × mol^−1^ × cm^−1^.

The differences in the given calculations of Fmoc group loading probably decrease with the increasing mass of TiO_2_@SiO_2_–(CH_2_)_3_–NH–Gly–Fmoc samples used for a measurement.

In the case of Fmoc group presence determination for Fmoc–Gly–OH in the solution used as the model sample, our calculation is almost the same regardless of the intensity of absorption band both at 289 nm, as well as 301 nm. The concentration of the Fmoc group was obtained at the level of 2 mmol/g.

The calculation of Fmoc-group loading at solid materials described in this work provides the information on how many active amino groups are present at these materials before modifications. The presented method is much simpler and cost-effective in comparison with the commonly used methods for amino groups determination. Additionally, presented method can be adopted for the characterization of nanomaterials.

## 3. Materials and Methods

### 3.1. Materials

All reagents and solvents were of analytical grade and used without purification. Sodium dodecyl sulfate (SDS), titanium (IV) isopropoxide (TTIP), sodium citrate dihydrate, tetraethyl orthosilicate (TEOS), 3-(aminopropyl)trimethoxysilane (APTMS), Fmoc–glycine (Fmoc–Gly–OH) used as a control sample, piperidine, *N*,*N*′-diisopropylcarbodiimide (DIC), 4-dimethylaminopyridine (DMAP) and tetrachloro-*p*-benzoquinone (*p*-chloranil) were purchased from Sigma-Aldrich (Poznan, Poland).

### 3.2. Synthesis of TiO_2_ Derivatives

#### 3.2.1. Synthesis of TiO_2_

Titanium precursor (TTIP) (9.678 g, 33.7 mmol) was added to 200 mL of 8-mmol/L SDS dissolved in water. The reaction mixture was stirred for 1 h in 90 °C until white precipitate was obtained. Subsequently, the obtained product was separated by centrifugation, washed with distilled water until complete removal of SDS and the dried on air.

#### 3.2.2. Synthesis of TiO_2_@SiO_2_

TiO_2_ (50 mg, 6.25 mmol) obtained in the procedure mentioned above was added to 100 mL of ethanol solution containing 30 mg of sodium citrate. The solution was vigorously stirred on magnetic stirrer and adjusted to pH = 9 using ammonia hydroxide. Then, 10 mL (44.83 mmol) of tetraethyl orthosilicate (TEOS) was added to the reaction mixture. After stirring for 24 h, the obtained white precipitate was collected by centrifugation, washed with ethanol and dried.

#### 3.2.3. Synthesis of TiO_2_@SiO_2_–(CH_2_)_3_–NH_2_

TiO_2_@SiO_2_ (100 mg) and 3-(aminopropyl)trimethoxysilane (APTMS; 0.7 mL, 4.00 mmol) were dissolved in 1.5 mL of toluene. Subsequently, the solution was transferred to a round-bottomed flask equipped with a reflux condenser and stirred at 120 °C for 24 h. The reaction mixture was cooled down to the room temperature, the obtained participate was washed with diethyl ether and dichloromethane and dried in a stream of nitrogen. The effectiveness of the amination reaction was confirmed based on the chloranil test [45].

#### 3.2.4. Synthesis of TiO_2_@SiO_2_–(CH_2_)_3_–NH–Gly–Fmoc

The mixture of TiO_2_@SiO_2_–(CH_2_)_3_–NH_2_ (40 mg) and Fmoc–glycine (Fmoc–Gly–OH) (10 mg, 0.0336 mmol) dissolved in methylene chloride: DMF, 1:1, (*v*/*v*) was cooled in an ice-bath to 0 °C. Then, 50 µL of *N*,*N*′-diisopropylcarbodiimide (DIC) and 0.5 mg of 4-dimethylaminopyridine (DMAP) were added to the solution and stirred overnight. After this time, the resulting residue was centrifuged and washed several times with DMF and dichloromethane until the absence of substrates. Obtained residue was dried in vacuum desiccator over P_2_O_5_ for 12 h at room temperature, resulting in 31 mg of the desired product.

### 3.3. Material Characterization

Transmission electron microscope (TEM) images were obtained on Tecnai G2 Spirit BioTWIN by FEI (Eindhoven, the Netherlands). Scanning electron microscopy (SEM) images were obtained on Hitachi S-3400N scanning electron microscope. Fourier-transform infrared spectroscopy (FT-IR) analyses were carried out using Bruker IFS 66 Spectrometer (Ettlingen, Germany).

The thermal analysis of the nanostructures was measured using Jupiter STA 449 F3 thermogravimetry connected to the QMS 403 C quadrupole mass spectrometer (Netzsch, Selb, Germany). The measurements were carried out in an inert gas (argon) atmosphere, from room temperature to 1000 °C, with a heating rate of 10 °C/min.

X-ray photoelectron spectroscopy (XPS) studies were carried out on PHI QUANTERA II spectrometer. The spectroscope is equipped with monochromatic AlKα source, the analyses were carried out with a spot diameter of 250 µm and in the binding energy range of C1s, O1s, N1s, Si2p and Ti2p spectra. Measurements were conducted with 20 eV pass energy, with the charge compensation controlled through low-energy electron and Ar^+^ ions flow by a flood gun.

The spectrophotometric measurements were determined by using UV-vis spectrophotometer, Perkin Elmer, Lambda 650 model (Shelton, CT, USA). The measurement was performed at 298 K using 1-cm-thick quartz spectrophotometric cuvettes in 20% (*v*/*v*) solution of piperidine in DMF at the 200–800 nm wavelength range.

### 3.4. The Procedure of Amino Group Loading on Nanoparticles

The dibenzofulvene–piperidine adducts is usually soluble in the reaction solvent. The occurrence of dibenzofulvene–piperidine in the solution after cleavage with piperidine gives the possibility to use the absorbance spectroscopy. This method is utilized to calculate the number of amino groups in the case of amino acid derivatives or surface coverage by amino groups in the case of NPs derivatives containing amino groups protected by Fmoc groups. In this work, we adopted the spectrophotometric measurements to determine the concentration of amino groups at the TiO_2_ NPs modified by Fmoc–Gly–OH derivative (TiO_2_@SiO_2_–(CH_2_)_3_–NH–Gly–Fmoc). Additionally, we calculated the amount of amino groups for Fmoc–Gly–OH used as a model sample according to the procedure described below.

About 2 mg of TiO_2_@SiO_2_–(CH_2_)_3_–NH–Gly–Fmoc sample Table 2 was weighted in a spectrophotometric quartz cuvette. Then, 3 mL of 20% (*v*/*v*) piperidine in DMF was added and the suspension was stirred for 20 min. After this time, the precipitate was left for 1 h to settle down at the bottom of the cuvette and then the absorption spectra were obtained. The estimation of the Fmoc group loading was conducted according to the equation [76]. The knowledge concerning the loading of Fmoc groups attached to NPs enables the estimation of amino groups load at the nanoparticle surface.

(1) $$Z=\frac{A\times v}{\varepsilon \times m\times l}$$

Z—Fmoc group loading (mol/g)
A—Absorbance value at the maximum absorption (nm)
v—Volume of solvent (dm^3^)
ε—Molar absorption coefficient (dm^3^×mol^−1^×cm^−1^ maximum absorption)
m—Sample weight (mg)
l—Length of the cell (1 cm)

## 4. Conclusions

In this study, the synthesis of TiO_2_, TiO_2_@SiO_2_, TiO_2_@SiO_2_–(CH_2_)_3_–NH_2_ core-shell structures was presented. The Fmoc–glycine (Fmoc–Gly–OH) was used as a modification agent of TiO_2_@SiO_2_–(CH_2_)_3_–NH_2_ to obtain TiO_2_@SiO_2_–(CH_2_)_3_–NH–Gly–Fmoc. All the obtained nanostructures were described using various analytical methods: transmission electron microscopy (TEM), Fourier-transform infrared spectroscopy (FTIR), thermogravimetric analysis (TGA) and X-ray photoelectron spectroscopy (XPS). Additionally, all of these measurements confirmed the effectiveness of each modification step. The deposition of Fmoc–glycine (Fmoc–Gly–OH) on the core-shell structure was used to develop the method for quantitative determination of Fmoc groups present at newly obtained materials. In this procedure, (Fmoc) groups were cleaved using 20% (*v*/*v*) solution of piperidine in DMF. This reaction leads to the formation of dibenzofulvene–piperidine adduct enabling the estimation of Fmoc groups loading by UV-vis spectroscopy. The comparison of different molar absorption coefficients—5800 and 6089 dm^3^ × mol^−1^ × cm^−1^ and 7800 and 8021 dm^3^ × mol^−1^ × cm^−1^ was used to calculate the loading of Fmoc groups at 289 nm and 301 nm, respectively. Furthermore, the presence of Fmoc groups in solution was calculated for Fmoc–Gly–OH used as the model sample. The described methodology is particularly useful for the determination of the number of amino groups in various nanomaterials.

## Figures and Tables

**Table 1:** Distribution of Si, N, O and Ti chemical states, based on the Si2p, N1s, O1s and Ti2p high-resolution XPS spectral analyses with the proposed deconvolution model for each studied TiO_2_@SiO_2_ nanoparticle functionalization step.

		BE/eV	TiO_2_@SiO_2_	TiO_2_@SiO_2_–(CH_2_)_3_–NH_2_	TiO_2_@SiO_2_–(CH_2_)_3_–NH–Gly–Fmoc
**Si2p**	SiO_2_	102.1	27	–	–
	Si–O–C	101.6	–	25.7	25.1
**O1s**	C–O, H_2_O	533.1	7.2	5.4	6.4
	SiO_2_, C=O	531.6	41.8	35.2	38.8
	SiO_2_–TiO_2_	530.2	16.7	12.2	9.2
	TiO_2_	528.8	4.5	2.7	1.1
**N1s**	N–C	398.5	–	12.1	12.7
	N=C	400.3	–	5.8	6.7
**Ti2p**	TiO_2_	459.2	2.8	0.9	–

**Table 2:** The absorbance maxima for TiO_2_@SiO_2_–(CH_2_)_3_–NH–Gly–Fmoc and Fmoc–Gly–OH at 289 nm and 301 nm.

Sample	TiO_2_@SiO_2_–(CH_2_)_3_–NH–Gly–Fmoc			Fmoc–Gly–OH		
	m (mg)	A@289 nm	A@301 nm	m (mg)	A@289 nm	A@301 nm
**(1)**	2.06	0.025	0.048	0.25	1.15	1.49
**(2)**	2.06	0.024	0.047	0.17	0.81	1.03
**(3)**	2.08	0.025	0.044	0.11	0.57	0.74

**Table 3:** Loading of Fmoc group (Z) for and Fmoc–Gly–OH calculated for 4 samples using a different value of molar absorption coefficient (ε).

	TiO_2_@SiO_2_–(CH_2_)_3_–NH–Gly–Fmoc		Fmoc–Gly–OH	
ε (dm^3^ × mol^−1^ × cm^−1^)	Z@289 nm (µmol/g)	Z@ 301 nm (µmol/g)	Z@289 nm (mmol/g)	Z@ 301 nm (mmol/g)
5800	6.3; 6.0; 6.2	–	2.38; 2.46; 2.68	–
7800	–	9.0;8.8;8.1	–	2.29; 2.33; 2.58
6089	6.0; 5.7; 5.9	–	2.27; 2.35; 2.55	–
8021	–	8.7; 8.5; 7.9	–	2.23; 2.27; 2.52
